# Can dialectical behavior therapy skills group treat social anxiety disorder? A brief integrative review

**DOI:** 10.3389/fpsyg.2023.1331200

**Published:** 2024-01-08

**Authors:** Mara Villalongo Andino, Katelyn M. Garcia, John A. Richey

**Affiliations:** Department of Psychology, Virginia Tech, Blacksburg, VA, United States

**Keywords:** social anxiety, dialectical behavior therapy (DBT), social anxiety (SA), interpersonal effectiveness, distress tolerance, emotion regulation (ER), mindfulness

## Abstract

The purposes of this brief integrative review are to identify and critically evaluate recent work in the area of Dialectical Behavior Therapy-Skills Group (DBT-SG) for Social Anxiety Disorder (SAD) with suicidal ideation (SI) and to suggest further how DBT-based skills may be applied to cognitive maintenance factors of SAD. Accordingly, we first evaluate the relevance of DBT in treating SI in other disorders. Second, we evaluate the relationship between SI and SAD, providing considerations for the complexity of comorbid disorders and presentations. Finally, we extend this knowledge to discuss considerations for the use of DBT-SG skills to target specific etiological and maintenance elements of SAD, with a focus on four themes (interpersonal effectiveness, mindfulness, emotion regulation, and distress tolerance). Overall, we conclude that DBT-SG may prove beneficial in reducing SI and symptoms in SAD that impact social and emotional functioning.

## Introduction

Social Anxiety Disorder (SAD) is a chronic and impairing disorder marked by a fear of one or more social or performance situations (American Psychiatric Association, 2013). SAD remains among the most common anxiety disorders, with an estimated current and lifetime prevalence of 7.1% and 12.1%, respectively (Kessler et al., 2005). SAD also has high rates of comorbidities, with 69 to 81% of affected individuals experiencing comorbid mental health conditions [i.e., major depression disorder (MDD), bipolar disorders, panic disorder, specific phobias, generalized anxiety disorder (GAD), alcohol use disorders, etc.; (Schneier et al., 1992; Magee et al., 1996; Weiller et al., 1996; Lecrubier, 1998; Kessler et al., 1999; Mennin et al., 2000; Kessler et al., 2005; Koyuncu et al., 2019)]. These comorbidities exacerbate symptom severity and contribute to poorer outcomes, including significant treatment resistance and decreased overall functioning (Koyuncu et al., 2019). SAD is further associated with increased reports of suicidal ideation (SI) and attempts when compared to other anxiety disorders (Sareen et al., 2005; Cougle et al., 2009; De La Vega et al., 2018). Furthermore, SI and attempts in SAD are more pronounced in incidence and severity, irrespective of comorbid depression (Herres et al., 2019). Previous research has noted comorbid diagnoses within this population exacerbate suicidal behaviors, thus underscoring the urgent need for new treatment avenues to address this pernicious aspect of the disorder (Weiller et al., 1996; Sareen et al., 2005; De La Vega et al., 2018; Koyuncu et al., 2019).

Currently, front-line treatments for SAD include Cognitive Behavioral Therapy (CBT), which is recognized as the most effective and widely recommended form of intervention (Gordon et al., 2014). Nevertheless, prior work suggests SAD is the anxiety disorder that is *least* responsive to CBT, as defined by remarkably modest remission rates, with up to 51% of cases remaining symptomatic following treatment completion (Juster and Heimberg, 1995; Springer et al., 2018). CBT’s limited success rates highlight that current “gold-standard” interventions may not engage core etiological and maintenance factors of SAD. Therefore, there is a pressing need to identify alternative intervention methods that may also target under-appreciated mechanisms of the disorder. Dialectical Behavior Therapy–Skills Group (DBT-SG) has demonstrated promise as a treatment for psychiatric disorders characterized by complex comorbidities and suicidal ideation and behaviors (Valentine et al., 2015). However, there have been no systematic considerations of DBT-SG for SAD or the extent to which hypothesized mechanisms of the disorder may fit this particular treatment approach. Accordingly, this brief review highlights a theoretical rationale for applying DBT-SG for SAD and considers the evidence for potential mechanisms by which this intervention may influence SAD course and severity.

Dialectical behavioral therapy (DBT) was first developed as an intervention for Borderline Personality Disorder [BPD; (Linehan, 1993)] and has also been used successfully in non-BPD populations (Valentine et al., 2015). “Full-model” DBT involves several components including individual therapy, group skills training, telephone consultation, therapist consultation, and team meetings (Linehan, 1993). However, research has documented that implementing DBT in community settings faces challenges like staff training, turnover, and financial constraints in seeking and sustaining services (Swenson et al., 2002; Carmel et al., 2014). Perhaps as a result, research has increasingly focused on implementing DBT Skills Group (DBT–SG) as a stand-alone treatment (Valentine et al., 2015). DBT-SG is led by two trained clinicians who meet with the group weekly and follow a protocol emphasizing four core skills: mindfulness, distress tolerance (DT), emotion regulation (ER), and interpersonal effectiveness (Linehan, 2014). Group meetings focus on covering assigned topics related to one of the four skills, followed by a discussion of the weekly homework (Linehan, 2014). These components address the core dialectic in DBT of *acceptance* and *change* while concurrently allowing the individual to pursue life values even while in psychological distress. Importantly, meta-analyses of DBT-SG studies highlight its efficacy as a stand-alone treatment in treating a variety of mental health concerns such as depression and other mood disorders, oppositional defiant disorder, eating disorders, attention-deficit hyperactivity disorder, BPD, with implementation in variety of clinical settings, such as prisons or medium security settings, community or psychology department clinics, and via telehealth (Valentine et al., 2015; Zalewski et al., 2021; Bean et al., 2022; Landes et al., 2022). DBT-SG effectively reduced suicidal behaviors and sustained that improvement over time while addressing comorbid disorders and symptoms (Sambrook et al., 2007; Soler et al., 2009; Long et al., 2011; Decker et al., 2019). Further, research found DBT-SG is similarly as effective to “full-model” DBT at reducing suicidal behaviors and non-suicidal self-injurious behaviors and reducing anxiety (Linehan et al., 2015).

## Suicidality and comorbidity in social anxiety disorder

The increased experience of suicidal ideation in SAD represents a potentially promising rationale and justification for considering the use of DBT-SG in this population (Sareen et al., 2005; Cougle et al., 2009; De La Vega et al., 2018). Research highlights individuals with SAD endorse SI, with 16% of individuals with SAD reporting SI in the previous month and 18% reporting a history of suicide attempts (Cougle et al., 2009; Bomyea et al., 2013). Moreover, Weiller et al. (1996), found SI worsens with comorbid diagnoses in this community. Their study revealed 8.5% of individuals with SAD had a history of SI, compared to 41.3% of those with comorbid SAD. Additional research found this relationship persisted after social anxiety symptoms were in remission and no longer met diagnostic criteria (Kessler et al., 1999). Researchers theorize this relationship stems from a lack of belongingness and perceived burdensomeness that those with SAD often experience, which are interpersonal factors associated with a higher suicide risk (Davidson et al., 2011; Silva et al., 2015; Chu et al., 2016; Buckner et al., 2017; Duffy et al., 2020). This association is thought to increase self-perceived social burden among those with SAD, leading to the belief that others would be “better off” without them (Duffy et al., 2020). Critically, DBT-SG has demonstrated effectiveness in reducing suicidal behaviors and sustaining that improvement over time (Decker et al., 2019). For example, Decker et al. (2019) investigated the application of DBT-SG for Veterans with suicidal ideation and found improvements in suicidal risk, SI, and a sustained improvement at a 3-month follow-up. Thus, DBT-SG offers a promising approach to address the under-appreciated risk of SI among those with SAD and could provide a more proactive approach in treating SI than other standardized treatments for SAD. In addition to the potential direct effect of DBG-SG on SI, extant research highlights the effectiveness of DBT-SG in addressing comorbid disorders, which may indirectly reduce suicidal behaviors and sustain improvement over time (Sambrook et al., 2007; Soler et al., 2009; Long et al., 2011; Decker et al., 2019). Available data suggest 69–81% of individuals with SAD endorse diagnostic criteria for another mental health diagnosis, which include other anxiety disorders (e.g., GAD or panic disorder), substance abuse disorders, and obsessive-compulsive or mood disorders, such as MDD (Schneier et al., 1992; Magee et al., 1996; Weiller et al., 1996; Lecrubier, 1998; Kessler et al., 1999; Mennin et al., 2000; Kessler et al., 2005; Koyuncu et al., 2019). A study by Soler et al. (2009) found secondary depression improved when implementing DBT-SG in a sample of individuals with BPD. These findings highlight DBT-SG’s utility in treating primary and secondary diagnoses; an aspect of DBT-SG that may be beneficial for treating SAD when considering the increased rate of comorbidities within this population.

## Social anxiety and dialectical behavior therapy - skills group

Another vital aspect of DBT is the four overarching themes within which these mechanisms exist and are actively practiced. A central point within this review is that DBT-SG may engage etiological and maintenance elements of SAD despite a lack of direct tests assessing the implementation of DBT-SG within this population. For example, cognitive maintenance factors of SAD that have received consistent empirical support include high social standards, perceived poor social skills, poorly defined social goals, lack of perceived control, negative self-perception, heightened self-focused attention, post-event rumination and avoidance, and safety behaviors (Clark and Wells, 1995; Hofmann, 2007).

### Interpersonal effectiveness skills

Regarding social standards, individuals with SAD frequently report believing they must achieve a high level of competency while concurrently underestimating their social abilities to meet that standard (Hofmann, 2007). Paradoxically, current research does not support the presence of social skills deficits in SAD but instead highlights that social performance abilities play a minimal role in many anxiety disorders (Clark and Arkowitz, 1975; Halford and Foddy, 1982; Rapee and Lim, 1992; Stopa and Clark, 1993). There is greater consensus regarding the importance of perceived social skills insofar as individuals with SAD appraise their social skills more negatively (Alden and Wallace, 1991; Rapee and Lim, 1992; Stopa and Clark, 1993). Alden and Wallace (1991) had socially anxious individuals rate themselves within a social interaction and found that socially anxious individuals demonstrated a negative bias in their performance and underestimated their social skill. Research has noted SAD treatment can boost how individuals perceive their performance, despite no objective improvements in performance (Newman et al., 1994). This discrepancy between the perceived social standard and perceived social abilities may result in a negative self-appraisal and a negative self-perception (Hofmann, 2007). Lastly, SAD can result in difficulty in setting, defining, and achieving social goals (Newman et al., 1994; Heinrichs and Hofmann, 2001; Hiemisch et al., 2002).

The interpersonal effectiveness skills in DBT-SG can target *perceived* deficits in social functioning and could address three core factors of SAD: high social standards and their ability to reach that standard, perceived poor social skills, and poorly defined social goals. This skill aims to maintain and improve relationships while maintaining self-respect (Linehan, 2014). This skill could help adjust their social standards and improve their goal-setting difficulties while helping them gain confidence in their social abilities and, as a byproduct, improve their negative self-perceptions and perceived lack of control. Notably, reviews on social skills emphasized that social skills training alone is ineffective in improving social skills in SAD (Ponniah and Hollon, 2008). However, the combination of the skills in DBT-SG for this population can more efficiently address the core aspects of the disorder.

### Mindfulness skills

Individuals with SAD utilize selective information processing, resulting in heightened self-focused attention. For example, social threats may lead to increased self-focused attention, detailed self-monitoring, and observation (Hirsch et al., 2003). SAD can also result in a lack of perceived control over adverse emotional and bodily reaction (Rapee and Lim, 1992; Zalewski et al., 2021), leading them to the perceived uncontrollability of emotional responses in social situations (Cloitre et al., 1992; Leung and Heimberg, 1996; Hofmann, 2007). Several skills within DBT-SG can concurrently address these factors, such as mindfulness, ER, and DT skills.

*Mindfulness* is a core DBT-SG skill that entails observing, describing, and participating fully in one’s actions and experiences in a non-judgmental manner with a focus on effective behavior (Linehan, 1993). As previously noted, there are currently no studies regarding the application of DBT-SG for SAD. However, several mindfulness-based interventions, such as Mindfulness-Based Stress Reduction and Mindfulness-Based Cognitive Therapy, can provide support for this component of DBT-SG as they have been efficacious in decreasing negative emotions and SAD symptom severity (Goldin et al., 2013; Strege et al., 2018). Within these interventions, the main objectives emphasize observing thoughts, emotions, and physical sensations in the present moment and critically doing so without judgment. Mindfulness is thought to target negative ruminative thought processes within SAD that, over time, may lead to behavioral avoidance of social situations. In turn, these can unlink the experience of social interaction from its typically reinforcing outcomes (Klemanski et al., 2017; Richey et al., 2019). Extant research highlights increases in mindfulness lead to increased executive and attentional control and as mindfulness skills improve so does the ability to control and direct attention (Lynch et al., 2006). Therefore, mindfulness could address the lack of perceived control and circumvent the heightened self-focused attention within SAD. These mindfulness skills in DBT-SG could engage SAD symptoms through the exact mechanisms of other mindfulness-based therapies.

### Emotion regulation (ER) skills

Several models of SAD emphasize the role of difficulties in ER, mainly decreased emotional awareness, emotional hyper-reactivity, and difficulty regulating the emotional experience (Clark and Wells, 1995; Rapee and Heimberg, 1997; Clark and McManus, 2002; Goldin et al., 2009). Individuals with SAD commonly employ two ER strategies: expressive suppression (ES) and cognitive reappraisal (CR). ES involves inhibiting outward emotional expression, potentially leading to the suppression of both positive and negative emotions and resulting in anhedonia (Erwin et al., 2003; Turk et al., 2005; Spokas and Heimberg, 2009; Watson and Naragon-Gainey, 2010; Werner and Gross, 2010). CR involves reframing one’s perspective to diminish or intensify emotional impact, thus altering the interpretation of the situation (Gross, 2002). While typically effective in reducing negative emotions, in SAD, this strategy is less successful, as individuals often doubt their ability to employ it effectively (Werner et al., 2011). The inability to regulate emotions before, during, and after a social interaction can contribute to anxiety, avoidance and impact social functioning (Erickson et al., 2014). Due to this difficulty in ER, evidence-based interventions for SAD should include an ER component.

ER skills within DBT-SG aim to improve emotional modulation and control, with the recognition that no one is ever in complete emotional control (Linehan, 1993). A key strategy is opposite action, which entails determining whether an emotion is justified, being exposed to the anxiety-inducing cue, and replacing the behavior elicited with new responses (Linehan, 1993). These skills broaden the cognitive response to the emotional experience and aid in changing perception of the emotional experience (Lynch et al., 2006). Improvements in ER skills can address several etiologic and maintenance factors within SAD, such as negative self-perception and lack of perceived control that will lessen by increasing self-esteem and a sense of control over emotional responses (Cloitre et al., 1992; Fernandes et al., 2022).

### Distress tolerance (DT) skills

DT pertains to the assessment and consequences of experiencing negative emotions. Individuals with SAD have lower DT which is associated with a heightened reactivity to stress, anxiety and distress (Keough et al., 2010). Moreover, this suggests ineffective coping mechanisms and results in concerted attempts to avoid negative emotions entirely. For example, Keough et al. (2010) theorized low DT might lead to perceiving anxiety symptoms as uncontrollable and intensify the reliance on avoidance and safety behaviors. Notably, low DT impacts treatment outcomes, as per Katz et al. (2017) who reported it predicted increased SAD symptom severity in therapy. This underscores the importance of addressing DT in treatment.

DT Skills in the DBT- SG focus on addressing two skills: crisis survival skills (i.e., tolerating painful events, urges, and emotions) and reality acceptance skills [i.e., minimize suffering by embracing a life different from their ideal (Linehan, 1993)]. These skills facilitate an improvement in conscious control over attentional processes, emotional and rational thinking integration, and a sense of unity with themselves (Linehan, 1993; Lynch et al., 2006). In SAD, this distress can intensify emotional reactions to social situations, resulting in hyperreactivity to negative emotions. Moreover, SAD can distort time perception, due to self-focused attention and post-event rumination, causing time to seem to pass slowly (Bar-Haim et al., 2010; Gross and Jazaieri, 2014). Lynch and colleagues (Lynch et al., 2006) found DBT may increase perceived control, reduce self-focused attention and improve control over attentional processes, fostering a sense of ownership and behavioral acceptance in individuals with SAD. Moreover, this skill fosters a radical acceptance of the current situation which could improve the negative self-perception within SAD.

### Combining skills in DBT-SG to address interrelated factors

Two final interrelated factors that maintain SAD are avoidance and safety behaviors (i.e., behaviors intended to reduce or hide anxiety) and post-event rumination [i.e., thoroughly reviewing social interactions, emphasizing anxiety and self-doubt (Clark and Wells, 1995; Heinrichs and Hofmann, 2001)]. Avoidance and safety behaviors disrupt social interactions by diverting attention to props or items during stressful social situations for temporary comfort (Clark and Wells, 1995; Heinrichs and Hofmann, 2001). These constructs produce a positive feedback loop that sustains social anxiety by preventing exposure despite repeated successful social encounters (Wells et al., 1995). Post-event rumination contributes to a negative self-perception, leading to anticipatory processing, which reinforces anxiety through past failures (Clark and Wells, 1995; Clark, 2001; Hofmann, 2007). Mindfulness skills could address these factors by changing emotion-linked automatic responses and fear, allowing individuals to acquire new responses (Lynch et al., 2006), and, thus, disrupting avoidance and safety behaviors. Moreover, as new automatic responses arise and individuals perceive themselves non-judgmentally, post-event rumination could improve, and the negative perception of themselves and their social skills could improve.

Similarly, ER skills interfere with the reliance on avoidance and safety behaviors by allowing people to engage in more effective behaviors that break down the association between the emotionally evocative stimulus and the unjustified emotional response (Gross, 1998). As the negative self-perception and lack of perceived control lessen due to the increased self-esteem and sense of control (Cloitre et al., 1992; Fernandes et al., 2022), this would lessen an individual’s need to engage in post-event rumination, especially if they are confident in their sense of control over the situation and in their behavior.

Interpersonal effectiveness would peripherally address these factors because, as high social standards, perceived poor social skills, and poorly defined social goals improve, so would the negative self-perception and lack of perceived control. Subsequently, the post-event rumination and the need for avoidance and safety behaviors would decrease. DT skills can address avoidance and safety behaviors by providing individuals with skills to tolerate anxiety without behavioral avoidance. Moreover, DT enhances control over attentional processes, allowing individuals with SAD to gain a sense of ownership and acceptance over their behavior and facilitating a detachment from the post-event rumination since individuals would feel less of a need to engage in that behavior when they can tolerate the interactions.

## Conclusion

SAD is a debilitating disorder that remains resistant to cognitive-behavioral treatment, leaving those impacted by the disorder with prolonged impairment in various aspects of day-to-day functioning. Specific characteristics of SAD that require particular consideration during treatment include its high comorbidity rate with other anxiety and mood disorders and pronounced SI. Within this review, we characterized DBT-SG as a potentially promising intervention for SAD with considerations for complex, comorbid disorders and maintenance factors that contribute to SI. This brief integrative review was motivated by the limited research on the use of DBT-SG in SAD, and focuses on the connecting the mechanisms of action in DBT-SG to the known mechanisms of psychopathology genesis and maintenance in SAD. We propose that, given the identification of SI in SAD in current research, DBT principles may also prove promising for this disorder. Given complications outlined in current research regarding the challenging nature of implementing DBT in community settings, we suggest that DBT-SG may be more suitable for SAD. Furthermore, elements of SAD that further perpetuate symptoms of the disorder, such as avoidance and difficulties with emotion expression, may be well addressed utilizing the four themes of DBT-SG: interpersonal effectiveness, mindfulness, ER and DT. Limitations of the current field include not only scarcity of social anxiety populations included in intervention work that utilizes DBT principles but also scarcity of treatment consideration of SI in SAD. This limits conclusions regarding differences in response to treatment based on severity of symptoms and complex presentations.

Limitations of the current review should thus be considered in light of the general limitations noted in the literature. Specifically, this review explored the themes within DBT-SG and their potential impact on SAD. However, discussions comparing these considerations for a range of SAD severity and in those with and without SI are limited due to the nature of the existing literature. Furthermore, this review primarily focused on adults with SAD. Therefore, discussion on the influence of DBT-SG may not be generalizable to other developmental periods in which SI may be pronounced (e.g., adolescence or late-life).

We propose the implementation of DBT-SG for SAD may prove beneficial in reducing SI and specific cognitive maintenance factors of the disorder, including but not limited to high social standards, perceived poor social skills, poorly defined social goals, lack of perceived control, negative self-perception, heightened self-focused attention, post-event rumination and avoidance, and safety behaviors. In addition to considering how these symptoms may be treated within each theme of DBT-SG, combining skills from multiple themes may prove useful in tackling complex symptoms. In light of the findings outlined here, future research may consider the direct impact of DBT-SG on SAD symptoms and maintenance, with specific considerations for measuring and monitoring SI.

## Author contributions

MV: Conceptualization, Investigation, Writing – original draft, Writing – review & editing. KG: Writing – original draft, Writing – review & editing. JR: Conceptualization, Supervision, Writing – review & editing, Writing – original draft.
